# Current and Emerging Therapies for Ocular Herpes Simplex Virus Type-1 Infections

**DOI:** 10.3390/microorganisms7100429

**Published:** 2019-10-10

**Authors:** Raghuram Koganti, Tejabhiram Yadavalli, Deepak Shukla

**Affiliations:** 1 Department of Ophthalmology and Visual Sciences, University of Illinois at Chicago, 1855 W. Taylor St, Chicago, IL 60612, USA; rkogan3@uic.edu (R.K.); yteja@uic.edu (T.Y.); 2 Department of Microbiology and Immunology, University of Illinois at Chicago, Chicago, IL 60612, USA

**Keywords:** herpes simplex virus, herpesvirus, keratitis, ocular therapy, antiviral, acyclovir

## Abstract

Herpes simplex virus type-1 (HSV-1) is a neurotropic, double-stranded DNA virus that can cause a wide variety of diseases, including many ocular pathologies. It is one of the leading causes of infectious blindness in the United States. Because of its ubiquitous nature and its potential to cause serious ocular maladies, there is a significant need for more effective antiviral therapies against ocular HSV-1. In this review, we discuss the lifecycle of HSV-1 as it pertains to corneal infections and the clinically approved as well as emerging treatments to combat HSV-1 infections. We also highlight some newly identified host targets for the antiviral drug development.

## 1. Introduction

Herpesviruses are a group of double-stranded DNA viruses that commonly infect humans [1]. There are three subfamilies of herpesviruses: alpha-, beta-, and gamma-herpesviruses [2]. The human viruses included in alpha-subfamily are herpes simplex virus type-1 (HSV-1), HSV-2, and varicella-zoster virus (VZV) [2]. The alpha-subfamily differs from its relatives in that it has the widest host range and a relatively short replicative cycle [2]. 

HSV-1 and -2 infect up to 90% of adults in the world [1]. HSV-1 alone infects 66% of the world’s population. Seropositivity for HSV-1 has been reported in 65% of Americans and more than 50% of Europeans [3,4]. Interestingly, the seroprevalence of HSV-1 in the developing world has been declining, with an estimated 14% reduction in the US in the past 30 years [3]. However, in some developing parts of the world, such as Latin America and sub-Saharan Africa, the prevalence of HSV-1 surpasses 90% [5,6]. On US soil, the prevalence among those under the poverty line is 52%, more than double the rate of those above the poverty line [3]. These epidemiological findings suggest that, from a macro perspective, improvements in economic development and public health may reduce the prevalence of HSV-1.

**Figure 1:**
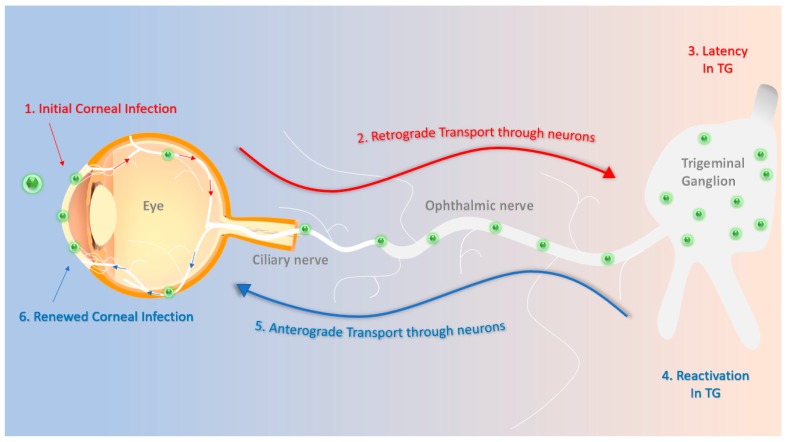
Schematics of herpes simplex virus type-1 (HSV-1) primary and recurrent infection. (1) The HSV-1 virions enter the cornea and initially replicate in the epithelium. (2) They then travel through the ciliary and ophthalmic nerves to the trigeminal ganglion in a retrograde fashion. (3) The virions establish a latent infection that can last for the lifetime of the host. (4) Stress-induced stimuli periodically reactivate the virus. (5) Reactivated virions travel through the ophthalmic and ciliary nerves in an anterograde fashion often to reach back to the site of initial infection. (6) HSV-1 re-infects the cornea, possibly leading to more pathologic symptoms, such as corneal scarring or neovascularization.

During a primary infection HSV-1 first infects the human eye at the corneal epithelium [7]. Once it successfully infiltrates the host cell at the corneal surface, it can engage in a lytic infection whereby it lyses the host cell and releases a multitude of virions to infect neighboring cells [8]. It then travels to the trigeminal ganglion via afferent neuronal cells and establishes a latent infection [9] (Figure 1). HSV-1 establishes an episomal latent infection: Instead of integrating its genome into the host’s DNA like retroviruses, it can store its genome in the nucleus of a host cell. HSV-1 can remain dormant or latent for the lifetime of the infected individuals [10]. During its latency, HSV-1 produces latency-associated transcripts (LATs) which maintain the integrity of the viral genome [10]. In many cases latent HSV-1 can reactivate and return to the site of the initial infection [10]. Episodes of reactivation worsen herpetic ocular disease and increase the chances of developing serious conditions, including significant vision loss or blindness [8]. 

Ocular HSV-1 infections can progress to a wide range of diseases that span the anatomy of the eye [1]. These include blepharitis, conjunctivitis, uveitis, retinitis, and keratitis which are the inflammation of the eyelids, conjunctiva, uvea, retina, and cornea, respectively [1,11]. Infections most often occur unilaterally, but immunosuppressed patients have an increased risk of bilateral infections [11]. Diseases of the outermost layers and the surface of the eye are the most common result of HSV-1 ocular infection, with one study reporting more than 50% of all ocular herpes infections occurring in the eyelids, conjunctiva, and cornea [12]. With regards to the risk of blindness, herpes stromal keratitis (HSK) is the most serious manifestation of ocular herpetic infections [13]. Patients with HSK experience recurring episodes of reactivation, and each recurrence further damages the cornea via processes such as opacification, neovascularization, and scarring [8]. Often the patients who suffer from HSK have to be continuously treated for a significant part of their lives. 

In this review, we discuss the lifecycle of HSV-1 as it pertains to corneal infections and the clinically approved as well as emerging treatments to combat HSV-1 infections. We also highlight some newly identified host targets for the antiviral drug development.

## 2. Viral Lifecycle: Attachment and Entry

HSV-1 is composed of many structural layers [14]. Located in the innermost space of the virus is an icosahedral nucleocapsid which encases the viral genome, which is a linear piece of double-stranded DNA packaged in a circular form [14]. Surrounding the capsid is the viral tegument, a layer of proteins and mRNAs which aid in the replication of viral DNA and inhibition of the host immune response [14]. A lipid bilayer envelope surrounds the tegument, and on the outside on the bilayer are about a dozen different glycoproteins that participate in entry and other important aspects of infection including viral release from infected cells [14]. 

**Figure 2:**
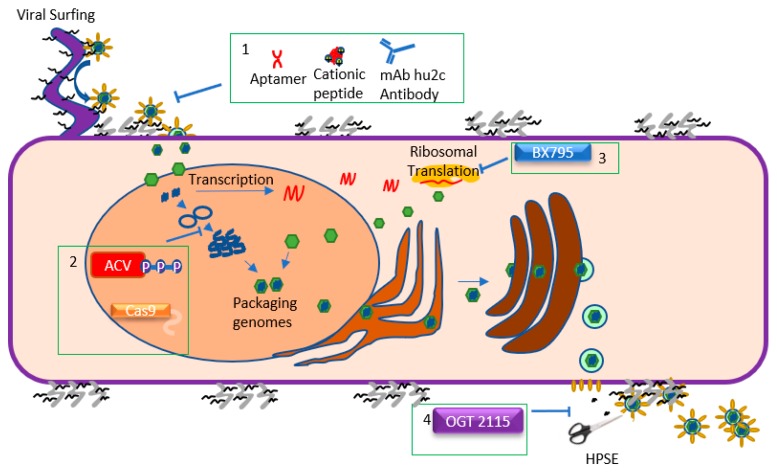
Schematics of the HSV-1 lifecycle and the steps targeted by antiviral agents. HSV-1 begins the entry process by attaching to heparan sulfate (HS) moieties located on proteoglycans on the cell surface. HSV-1 can also attach to HS chains on filopodia and engage in “viral surfing” along the filopodia to the cell surface. Once the HSV-1 glycoproteins have attached to their appropriate receptors, the viral envelope fuses with the host cell membrane, releasing the tegument and nucleocapsid into the cytoplasm. Viral entry can be inhibited (**Box 1**). Once in the cell, the capsid travels to the nucleus and injects the viral genome into it. HSV-1 then undergoes a process of circularizing, concatemerization, and packaging its genome into new capsids. During this period, the virus also transcribes mRNA and translates it, creating new proteins. Acyclovir and CRISPR/Cas9 inhibit vDNA replication, while BX795 impedes viral translation (**Boxes 2 and 3**, respectively). Once the new virions are produced, they travel from the nucleus through the ER and the Golgi apparatus, acquiring an envelope in the process. The enveloped virions then bud from the cell at specific locations. Presence of HS chains on the cell surface can trap virions, which is why HSV-1 upregulates heparanase (HPSE) during the later stages of infection to cleave the chains and promote egress. Viral egress is therefore inhibited by OGT 2115 (**Box 4**), a drug which inhibits the enzymatic activity of HPSE.

HSV-1 possesses four viral glycoproteins (gB, gD, gH, and gL) that are essential for viral entry and two other glycoproteins (gC and gK) are known to regulate viral attachment and membrane fusion, respectively [15,16,17]. Entry is a stepwise process, with the first event being the interactions of gB, gC, or both with heparan sulfate (HS) chains on heparan sulfate proteoglycans (HSPGs) located on the exterior surface of the target cell [18] (Figure 2). Next, gD interacts with one of four known gD receptors: nectin-1, nectin-2, herpesvirus entry mediator (HVEM), and 3-O-sulfated HS (3-OS-HS) [15]. The binding of a gD receptor activates a signaling pathway in which a fusion complex made up of gB, gH, and gL is recruited to the host cell membrane [19]. These interactions create a fusion pore on the target cell which allows the tegument layer and nucleocapsid to enter into the host cell [19]. Ocular cells express at least two or more of the known entry receptors [20,21,22].

## 3. Viral Lifecycle: Replication and Translation

After the nucleocapsid enters the host cell, it translocates to the nucleus along microtubule tracks with help from the cellular motor protein dynein [23]. The viral tegument proteins gradually release from the capsid during their trip to the nucleus [19]. Once it reaches the nuclear membrane, the nucleocapsid does not enter the nucleus itself but instead releases the viral genome into the nucleus through a nuclear pore [24]. The attachment of the capsid to the nuclear membrane is mediated in part by the tegument protein VP1/2 and a complex of the nucleoporins Nup358 and Nup214 [25,26,27]. Once safely inside the nucleus, the 152 kb linear genome proceeds to circularize, and the host RNA polymerase II transcribes viral mRNA to be transported to the ribosomes for protein synthesis in the cytoplasm [24].

HSV-1 transcripts are derived from three different kinetic classes of genes: Immediate-early (IE), early (E), and late (L) [24]. These groupings of genes are also known as the α, β, and γ genes, respectively, and are transcribed by the host RNA polymerase II [28]. Furthermore, these classes of HSV-1 genes are transcribed temporally: Each class regulates the expression of the subsequent one [24]. The transcription and subsequent translation of the six IE genes, ICP0, ICP4, ICP22, ICP27, ICP47, and US1.5, create proteins which regulate the expression of the other two classes of viral genes [29]. The IE genes also serve key roles in evading the host immune system. For example, ICP0 is an ubiquitin ligase which induces the degradation of host genes which stimulate interferon production [29,30]. ICP47 is unique in that it does not regulate E genes but solely helps HSV-1 evade CD8+ T cells [31]. The other five IE genes upregulate the transcription of E genes, and E genes regulate the expression of both IE and L genes, downregulating the former and upregulating the latter [Many of the E genes, such as ICP8, thymidine kinase (TK), and UL9, function in viral DNA synthesis].

DNA replication requires certain E genes for initiation and L genes for termination, and disruption of either process results in a failure to successfully replicate the viral genome [32,33]. During the replication of viral DNA, the viral DNA polymerase begins replication at one of three origin sites [33]. The polymerase creates long concatemers of DNA, each containing many replication forks [33]. The net effect of the process is the creation of a branched network of DNA which is then cut into individual viral genomes by proteins produced from L genes and placed into capsids, the assembly of which also occurs in the nucleus [33]. Once the nucleocapsid has its genomic payload inside, it escapes from the nucleus via budding or through a nuclear pore [34].

The expression of L genes, which include VP16, VP22, gB, and gC, are divided into two classes γ1 and γ2 [35]. The products of γ1 genes function in DNA replication, such as the cleaving the DNA concatemers mentioned previously [35]. Proteins derived from γ2 genes consist primarily of structural proteins which play key roles in assembling viral progeny and facilitating egress from the cell [35]. Certain IE proteins also display functions similar to L proteins. For example, the IE protein ICP27 is necessary for the packaging of ICP0 and ICP4 into new virions [36].

## 4. Viral Lifecycle: Assembly and Egress

While its genome and nucleocapsid are first assembled in the nucleus, HSV-1 finishes its assembly process in the cytoplasm [37]. During the process of leaving the nucleus, the virus envelopes itself with a lipid monolayer derived from the inner nuclear membrane [38]. The envelopment is mediated by the viral L genes UL31 and UL34 [38]. Additionally, gK, gM, and UL11 are believed to be implicated in the initial or primary envelopment of HSV-1 [39,40,41]. While not fully understood, it is thought that the virus then fuses with the outer nuclear membrane, releasing the nucleocapsid into the cytoplasm but losing the envelope it acquired from the inner leaflet of the nuclear membrane [42]. The crossing of the perinuclear space appears to be mediated by either gB or gH [43]. The virion then acquires another envelope and tegument layer in the cytoplasm through contact with a vesicle derived from the trans-Golgi network [37]. The vesicle also contains glycoproteins needed for egress from the host cell and entry into new cells [37]. The process of organizing assembly from the capsid, tegument proteins, the envelope, and glycoproteins relies upon a dense web of interactions between these components, many of which are described in detail in this review by Owen et al. [44].

Once it acquires an envelope again, HSV-1 travels to the cell membrane [42]. The virus modifies host protein myosin Va in order to penetrate the actin cortex and reach the cytoplasm unhindered [45]. From there, the virus egresses via exocytosis and emerges from the cell as a mature, enveloped virion [46]. The direction of egress of HSV-1 may be dependent on the glycoproteins gE and gI as the loss of either protein results in the accumulation of viral progeny at the apical surface of cells [47]. HSV-1 can escape from the apical surface of infected cells to infect nearby cells or from the lateral surface to infect adjacent cells with the help of cell-cell junctions [37,47].

Interestingly, HSV-1 also manufactures virions that only contain a tegument layer and envelopes, devoid of a nucleocapsid and DNA [48]. These light (L) particles may aid viral infection by adding more tegument proteins to the cells that they enter [48]. Another interesting part of viral release is the exploitation of host enzymes heparanase (HPSE) and Cathepsin L [49,50,51]. The enzymes are transported to the surface of infected cells where they remove heparan sulfate, which ensures that exiting virions are not trapped and allows for a smoother detachment or release of the virions. Inhibition or deletion of these enzymes results in significant reduction in HSV release from cells, and it was shown specifically for corneal epithelial cells [49].

## 5. Challenges and Recent Progress in Controlling Ocular Herpes

Ocular herpes is currently an infection for life. For a very long time, finding a cure for HSV has been a significant unmet need for physicians and scientists alike. Currently, with the exception of VZV, there is no vaccine for herpesvirus infections [52]. Also, the currently approved therapies to control ocular herpes provide limited efficacy and often have to be combined with steroids to reduce symptoms especially during the recurrent cases of HSK. In general, current treatment modalities reduce the symptoms only by a few days [53,54,55,56]. Likewise, long-term use of steroids has its own serious side effects, which includes increase in intraocular pressure and possible onset of secondary glaucoma [11,57,58]. While the clinically approved drugs, mainly nucleoside analogs, are clearly beneficial (Table 1), they also suffer from their share of pitfalls. Development of resistance against the most commonly used drug, acyclovir, is common and long-term use of it is known to cause renal toxicity [59,60]. Thus, there is a great need to develop new antiviral therapies for herpesvirus infections. Both currently approved as well as some emerging therapies are discussed below.

## 6. Approved Therapy: Acyclovir and Other Nucleoside Analogs

Acyclovir (9-[2-hydroxyethoxymethyl]guanine) is a purine nucleoside analog which inhibits the replication of HSV-1 [73]. Currently, it is the main treatment for HSV-1 infection [57,63,74]. Acyclovir can be administered topically, orally, or intravenously to patients [62]. There appears to be no significant differences in patient outcomes between the topical and oral formulations of acyclovir [62,74]. However, given its lower bioavailability on ocular surface, topical acyclovir is not recommended for ocular therapy in the US. Multiple studies report that acyclovir has beneficial effects when it is administered therapeutically or even prophylactically to prevent recurrent infections [62,74,75,76,77]. There exist multiple analogs for acyclovir, including valacyclovir, famciclovir, ganciclovir, and penciclovir [62,67]. While they possess similar mechanisms of action, these analogs differ in their bioavailability and thus require distinct dosing strategies [62,67].

Once the cell takes in acyclovir, it is phosphorylated by the viral protein thymidine kinase [63,75]. The acyclovir monophosphate is then phosphorylated twice more by host enzymes, converting it into its active form acyclovir triphosphate [63,75]. Acyclovir trisphosphate competes with deoxyguanosine trisphosphate (dGTP) to be incorporated into a new strand of vDNA, and its lack of a 3’ hydroxyl group terminates DNA replication prematurely [62,63,75]. Because DNA polymerase has a higher affinity for acyclovir triphosphate than dGTP, it is an efficient treatment option for HSV-1 [63,78]. Notably, these effects are only seen in infected cells [78]. Uninfected cells do not experience the inhibitory effects of acyclovir since the initial step of phosphorylating base acyclovir does not occur to a significant degree in uninfected cells [78].

There are two main weaknesses of acyclovir in the treatment of ocular HSV-1 infections. The first is that, since it is a nucleoside analog, it does not inhibit the synthesis of viral proteins directly [79]. Although the expression of certain late genes requires the successful completion of DNA replication, it is an imperfect way of reducing the production of viral proteins. Secondly, acyclovir is susceptible to drug resistance. Many cases of drug resistance have been reported, and immunocompromised patients appear especially vulnerable to developing resistant HSV-1 infections [76,80,81,82]. The mechanism by which resistance occurs usually involves a deficiency in thymidine kinase [83]. HSV-1 either does not produce a thymidine kinase or generates one that cannot interact with acyclovir [83]. One way to mitigate aforementioned problems is to administer acyclovir topically, at the site of infection, rather than a systemic administration. This not only reduces emergence of resistance but also alleviate toxic side effects associated with long term use [62]. However topical formulations have proved to be not as effective given the low retention time on the ocular surface [70]. To address this problem, recently our lab has shown that administration of acyclovir through a carbon-based drug delivery platform termed DECON can increase the efficacy of topical acyclovir while conferring protection for a long period of time [84]. DECON is both muco-adhesive and non-toxic. It loads high volume of drugs and delivers them to mucosal surfaces in a sustained fashion, making it a novel alternative to conventional dosing systems [84].

## 7. Emerging Therapy: BX795

BX795 is a well-known inhibitor of PDK1, and its downstream effects result in the inhibition of many other kinases including TANK-binding kinase 1 (TBK1), Aurora B kinase, and IĸB kinase (IKK) [85]. Unlike many other current treatments for ocular HSV-1 infection, it is not a nucleoside analog [11,57]. Instead, BX795 inhibits Akt phosphorylation at Ser473 and prevents the downstream hyperphosphorylation of the eukaryotic translation initiation factor 4E-binding protein 1 (4E-BP1) in a TBK1- and PDK1-independent manner [86]. Stimulation of Akt signaling upon viral entry and subsequent activation of mTORC1 have been shown to increase viral protein synthesis, so the inhibition of this pathway is an effective way to impede viral activities within the cell [87,88]. Importantly, the therapeutic concentration of BX795 does not appear to be toxic to human corneal epithelial cells [86]. Both in vitro and in vivo murine experiments with BX795 demonstrate a significant reduction of HSV-1 with little to no adverse effects [86]. Ex vivo experiments on human and porcine corneas again reveal a blockage of infection without apparent toxicity [86]. It should be noted that BX795 does not exhibit any synergy with other common antivirals, including trifluorothymidine and acyclovir [86]. 

In total, these results suggest that BX795 may be a promising antiviral against ocular HSV-1 infection and other viruses which utilize the Akt pathway to promote viral protein synthesis. Prophylactic studies of BX795 on ocular HSV-1 infections are currently underway. In addition, the precise mechanism by which BX795 acts on Akt and the long-term effects of BX795 treatment still must be elucidated.

## 8. Emerging Therapy: Nucleic Acid Aptamers

A nucleic acid aptamer is a short oligonucleotide with the ability to bind to a wide variety of molecular targets [89]. Their sizes range from 20 to 100 nucleotides, and they can bind to targets as small as ions and as large as entire organs [90]. The key strength of aptamers is their diversity in both structure and function [89,90].

One study reported that two RNA aptamers, designated aptamer-1 and aptamer-5, were able to bind to the HSV-1 protein gD with such high specificity that they could distinguish it from the HSV-2 gD [91]. Aptamer-1 in particular could inhibit the interaction between gD and its receptor HVEM with an EC50 of only 60 nM [91]. These findings were supported by a plaque assay which demonstrated that aptamer-1 could inhibit HSV-1 entry into Vero cells with a Ki of 0.8 [91]. Furthermore, the authors were able to use the functional region of aptamer-1 to create a smaller aptamer of 44 nucleotides, compared to 113 for the original, which was able to inhibit HSV-1 equally well as its parent aptamer [91].

In another study, an RNA aptamer for HSV-2 gD was isolated and found to exhibit significant antiviral activity [92]. It interfered with the interaction between HSV-2 gD and its receptor nectin-1 with an IC50 in the range of 20 to 50 nM [92]. Just as the authors above, they were able to isolate the functional segment of their aptamers which turned out to be a five-nucleotide motif [92]. 

While they possess many strengths over traditional therapeutic options, RNA aptamers display low stability and possess high manufacturing costs [93]. The solution to these drawbacks may be DNA aptamers which are more stable and cheaper to produce than their RNA variants [93]. By mimicking the structure of the mini aptamer-1 discussed before but incorporating thymine instead of uracil, the authors of one study were able to produce a DNA aptamer with a lower entropy value, suggesting that it is more stable at high temperatures [93]. The DNA aptamer was able to bind to its HSV-1 gD successfully (EC_50_ = 2 µM, *K*_d_ = 53.92 nM), but it displayed a slightly lower affinity than the RNA version [93]. Immunoblotting and plaque assays confirmed that the DNA aptamer inhibited viral entry and the overall HSV-1 infection in in vitro, ex vivo, and in vivo models [93]. Furthermore, the DNA aptamer exerted antiviral effects in prophylactic, neutralization, and therapeutic experiments [93].

The lack of toxicity reported with these aptamers and their ability to inhibit HSV-1 and HSV-2 infections with great specificities and low concentrations make aptamers an attractive treatment option for these types of alphaherpesviruses. Given that their potential is virtually unlimited with regards to their structure and target molecules, aptamers present a great opportunity for developing therapies, not just against HSV, but against many pathogens.

## 9. Emerging Therapy: Cationic Peptide Therapies

3-*O*-Sulfated HS is a known entry receptor for HSV-1 gD [19]. The inhibition of this interaction between gD and 3-*O*-sulfated HS is one mechanism by which viral entry can be impeded [8]. One of the earliest studies of the use of cationic peptides as antiviral agents was performed in 2001. The authors developed a FGF4 signal peptide modified with a highly cationic tetramer at the N-terminus [94]. The resulting peptide, given that BSA was present in the serum at sufficient concentrations, demonstrated the ability to block HSV-1 entry by binding to free virions [94,95]. Another study reported that using small peptides with differing cationic charge distributions could bind to HS and block gD [96]. The first class of 12-mer peptides, which were designated G1 peptides, had alternating charges, while the second class, G2 peptides, had many adjacent positive charges [96]. Both classes demonstrated the ability to block HSV-1 viral entry in vitro and in vivo [96]. These results highlight the importance of HS in HSV-1 entry and how the annulment of this pathway can significantly reduce viral infection. 

The G2 peptide therapy was further investigated in conjunction with acyclovir in another study. When comparing the effects of prophylactic treatment of G2, acyclovir, or both antivirals, all showed significant reductions in viral entry and replication with the greatest decrease found in the G2-acyclovir group [97]. The synergistic effects of the combination of drugs may be due to the membrane penetration achieved by the G2 peptide which could increase the amount of acyclovir that enters the cell [97]. The combined effects of a viral entry inhibitor and a nucleoside analog make the pairing a potentially more efficient therapy than the current recommendations of acyclovir alone.

A more stable version of G2 was developed by adding cysteine to its C-terminus, designated G2-C, that could be applied onto a contact lens [98]. When applied to the cornea, the G2 peptide was released over an extended period of time [98]. The G2-lens combination was effective in blocking HSV-1 entry in both ex vivo and in vivo corneal models [98]. This study demonstrates the translational relevance of G2 peptide therapies and how they may be a better alternative to administering antiviral drugs on a daily or weekly basis. G2 applied onto contact lenses may improve adherence to therapies, reducing the risk of recurring episodes of infection, without lowering the efficacy of the treatment. 

G1 and G2 peptides are not the only small peptide therapies which have shown to be effective treatments against HSV-1. A synthetic variant of the theta-defensin family, retrocyclin-2, was used to treat HSV-1 keratitis in a murine model [99]. When used prophylactically, retrocyclin-2 was shown to be effective in protecting the cornea from infection [99]. However, in a mouse already suffering from keratitis, retrocyclin-2 showed limited benefits [99]. Because retrocyclins inhibit viral entry and not replication, they may be of limited effectiveness when treating infections [99]. However, further work should be done to observe if other members of the theta-defensin family can exert stronger antiviral effects than retrocyclin-2. 

Another peptide derived from the HIV Tat protein, TAT-Cd, was tested in both in vitro and in vivo models against HSV-1 infections [100,101]. When given at a sufficient concentration of 1 mg/mL and up to four hours post infection, TAT-Cd was reported to reduce keratitis present in the cornea [100]. It was able to be delivered in a variety of manners (artificial tears, PBS, methylcellulose, and aquaphor cream), giving it great versatility in terms of reaching the cornea [100]. However, because TAT-Cd was shown to be ineffective when administer 24 hours post-infection, it may not be the best treatment option in its current form [100]. 

The peptides discussed above are all highly cationic molecules that demonstrate some degree of inhibiting ocular HSV-1 infection in in vivo models. In addition, the G2 peptide has been shown to synergize with acyclovir treatment. These promising results illustrate the protective effects of cationic peptides against HSV-1 infection. Further investigation into more varieties of cationic peptides may shed light onto even better therapies, both in isolation and combination with other mainstream drugs, against HSV-1 infections.

## 10. Emerging Therapy: CRISPR/Cas9 System

Currently, the only approved antiviral treatments for HSV-1 infections involve nucleoside analogs which inhibit the replication of viral DNA [102]. Corticosteroids can be used to reduce inflammation, but they do not directly inhibit the viral infection (Table 1). During a latent HSV-1 infection, such treatments are ineffective as the virus is not actively replicating its genome with its DNA polymerase. The efficacy of the even the newer treatments mentioned above to treat a latent HSV-1 infection has also not been demonstrated. This niche of treating a latent HSV-1 infection may be fulfilled by clustered regularly interspaced short palindromic repeats (CRISPR)/Cas9 technology.

The CRISPR/Cas9 system was derived from the adaptive immune systems of the bacteria and archaea domains [103]. In these microorganisms, the CRISPR/Cas system utilizes RNA peptides to degrade the genetic material of phages and other viruses [103]. In 2013, researchers adapted the CRISPR/Cas system for use in eukaryotic cells, and the technology has been steadily improving ever since [104,105]. In the newly engineered system, the bacterial Cas9 nuclease and a guide RNA (gRNA) are transfected into cells [104,105]. The guide RNA is made up of two components: A tracr RNA which binds to Cas9 and a crispr RNA (crRNA) that binds to a specific sequence of DNA [104,105]. The gRNA recognizes a target sequence on the host genome and brings the Cas9 protein to that location [106]. Once there the Cas9 nuclease breaks both strands of DNA, and the repair pathways of the host cell activate [106]. However, since the host repair mechanism makes many mistakes, the target gene site is mutated in the process [106]. 

The CRISPR systems have been shown to modify the genomes of HSV-1 and even clear latent Epstein-Barr virus (EBV), a member of the gammaherpesvirus subfamily, in vitro [107,108,109,110]. Recently, many strides have taken place in the usage of CRISPR to inhibit HSV-1 infections. In 2016, Roehm et al. utilized CRISPR/Cas9 technology to induce mutations in exon 2 of the ICP0 gene, an IE gene [111]. The loss-of-function mutations in ICP0 caused a sharp decline in the ability of newly produced viruses to replicate as measured by a plaque assay [111]. In addition, the authors demonstrated that the editing of ICP0 and the resulting decline in infectivity could take place in a human cell line, specifically the oligodendroglioma cell line TC620 [111]. CRISPR/Cas9 may have potential for prophylactic treatments as well: the TC620 cells were protected from HSV-1 infection when treated with lentiviruses containing Cas9 and gRNA prior to infection [111].

In another study published in 2016, van Diemen et al. significantly inhibited HSV-1 replication after transfecting Vero cells with one of twelve gRNAs with greater effects observed for the inhibition of essential genes (UL8, UL29, UL52) [106]. Transfecting cells with 2 gRNAs further impeded viral replication, and if two gRNAs targeting essential genes were used, viral replication was abolished: A six-log reduction in viral progeny was observed [106]. With regards to latent HSV-1 infections, the CRISPR/Cas9 system could not efficiently edit quiescent HSV-1 genomes [106]. However, it was effective in causing mutations in newly made viral DNA from reactivated HSV-1, further supporting the ability of CRISPR/Cas9 to confer prophylactic benefits to cells [106]. 

While no studies report the removal of latent HSV-1 genomes from host cells as of yet, there exists great potential to modify the CRISPR/Cas9 system to better target latent infections. Because attempts to edit latent genomes were only performed in Vero cells, it is possible that other model systems differ in the degree to which CRISPR/Cas9 functions. Given that researchers have eliminated latent EBV infections from cells in 2014, it seems likely that similar results can be accomplished with HSV-1 [108]. Current systems are able to reduce HSV-1 replication significantly in both infected cells and cells experiencing a reactivation of HSV-1 from quiescence. Future in vitro and in vivo studies are needed to examine the effects of CRISPR/Cas9 on HSV-1 infections and optimize the system. Current work suggests that it may be an option to inhibit viral replication in a TK-independent manner and holds promise for the elimination of latent HSV-1 genomes as well.

## 11. Emerging Therapy: OGT 2115

Heparanase, an endo-β-D-endoglycosidase capable of cleaving heparan sulfate, is important for HSV-1 release from infected cells including those from the cornea [49]. HSV-1 upregulates HPSE during the course of infection [49]. In vitro and in vivo experiments reveal that overexpressing HPSE leads to an increase in viral egress, and the reverse is true for cells given shRNA targeting HPSE [49]. Mechanistically, HPSE is upregulated and translocated to the nucleus upon HSV-1 infection. It increases the nuclear translocation of NF-κB in the process and reduces interferon expression, inhibiting downstream antiviral pathways [50]. It also increases the expression of pro-inflammatory factors such as IL-1β and IL-6 which may partly explain why overexpression of HPSE worsen corneal disease in vivo [50]. 

OGT 2115 has been characterized as an inhibitor of HPSE enzymatic activity [112]. Treatment with OGT 2115 two hours post infection has been shown to impede the expression of pro-inflammatory factors, inhibit plaque formation, and reduce the expression of gB and nuclear translocation of NF-κB [50]. In an ex vivo porcine cornea model, administration of OGT 2115 resulted in the clearance of GFP-HSV-1 as confirmed by immunofluorescence imaging and quantification of the results [50]. 

Similar to BX795 and the cationic peptides discussed previously, OGT 2115 is one of the few therapeutic options for HSV-1 infection that does not exert its effects by inhibiting viral replication. In this case, it appears to hinder viral egress via the inhibition of HPSE. To date, only one study examines the effects of OGT 2115 on HSV-1 infection. Further work must be done to investigate how OGT 2115 inhibits HPSE and whether it could be a suitable candidate for clinical trials in humans.

## 12. Emerging Therapy: Antibodies

Current antiviral therapies often lead to drug resistance in patients whose immune systems are compromised. The usage of antibodies as antiviral agents may be an effective alternative to traditional therapies. In 2013, Krawczyk et al. developed a humanized monoclonal antibody, designated mAb hu2c, against the viral glycoprotein gB [113,114,115]. mAb hu2c proved to be effective in neutralizing wild-type and drug resistant strains of HSV-1, blocking the spread of the virus between cells [113]. The positive in vitro results were further supported by in vivo murine models where both prophylactic and therapeutic treatment with mAb hu2c conferred protection against herpes simplex encephalitis in immunodeficient mice [113]. 

In another study by the same group, the authors focused on the efficacy of mAb hu2c in ocular models. They demonstrated that systematic administration of mAb hu2c reduced the viral load in mouse eyes when given between 24 hours pre-infection and 56 hours post-infection [116]. However, therapeutic treatment six days post-infection of mAb hu2c delivered systemically to mice did not change the viral load present [116]. These results highlight the time-sensitive nature of antibody this treatment. In addition, mAb hu2c was shown to reduce reactivation rates of HSV-1 infections in the trigeminal ganglia of mice [116].

Taken together, these findings demonstrate the power of humanized antibodies for treatment of ocular HSV-1 infections. Humanized antibodies may be another layer of defense, especially for vulnerable, immunocompromised patients with drug-resistant strains of HSV-1.

## 13. Discussion

Acyclovir and its relatives are no doubt effective treatments for ocular HSV-1 infections. However, their extensive downfalls combined with ubiquitous HSV-1 infections create major gaps in treatment options. Furthermore, the nucleoside analogs are the only major class of drugs currently approved for use in patients. Other options are needed to reduce the frequency and intensity of ocular infections, especially since viral resistance to acyclovir is on the rise. In this review, we discussed alternative treatments that target different phases of HSV-1 infection: Aptamers, retrocyclin 2, and antibodies for attachment; G1, G2, and other cationic peptides for entry; nucleoside analogs and CRISPR/Cas9 for DNA replication; BX795 for protein synthesis; and OGT 2115 for egress. These therapies demonstrated significant antiviral effects during their use in various in vitro, in vivo, and ex vivo experiments. 

It may be possible for drugs such as BX795 and OGT 2115 to treat ocular HSV-1 infections in patients who are resistant to acyclovir therapy. The HSV-1 strain would have to acquire resistance to three different mechanisms of treatment in order to replicate and infect other cells successfully. Additionally, aptamers, antibodies, and gRNAs have the potential to target and inhibit a tremendous range of viral proteins. Results with gB and gD have been quite positive in antibody and aptamer therapy respectively, and targeting other viral proteins or including multiple targets may well result in a greater reduction in viral load. The flexibility of these therapies offers them much room to grow and become optimized in the future. Additional work needs to be done in translating these emerging antiviral agents into real therapeutic and prophylactic treatments for patients. If successful, these alternative therapies would fulfill a vital niche in the quest to treat or prevent ocular HSV-1 infections.

## Figures and Tables

**Table 1:** List of approved drugs for ocular HSV-1 infections in the USA. Only the topical gels are approved specifically for the treatment of keratitis [61]. The name of the drug, its mechanism, how it is given, common dosages, and references are provided for each entry in the table.

Name	Mechanism of Action	Application	Recommended Dosage	Reference
Acyclovir	Guanosine Analog	Systemic	400 mg 3-5 times/day	[62,63,64]
Ganciclovir	Guanosine Analog	Topical	1 drop of 0.15% gel 5 times/day	[65,66]
Valacyclovir	Guanosine Analog	Systemic	500 mg 2 times/day	[67,68]
Famciclovir	Guanosine analog	Systemic	250 mg 2 times/day	[61,69,70]
Trifluridine	Thymine Analog	Topical	1 drop of solution 9 times/day	[61,71]
Corticosteroids	Anti-inflammatory agent	Systemic, topical	Frequency based on severity of Inflammation	[72]
